# Die Behandlung der proximalen Humerusfraktur im Kindes- und im Jugendalter: Konsensusbericht der Sektion Kindertraumatologie der DGU

**DOI:** 10.1007/s00113-024-01440-2

**Published:** 2024-05-30

**Authors:** Hauke Rüther, Peter C. Strohm, Peter Schmittenbecher, Dorien Schneidmüller, Sektion Kindertraumatologie (SKT) in der Deutschen Gesellschaft für Unfallchirurgie (DGU), Jörn Zwingmann

**Affiliations:** 1 Klink für Unfallchirurgie, Orthopädie und Plastische Chirurgie, Universitätsmedizin Göttingen, Robert-Koch-Str. 40, 37075 Göttingen, Deutschland; 2 Klinik für Orthopädie und Unfallchirurgie, Klinikum Bamberg, Bamberg, Deutschland; 3 Karlsruhe, Deutschland; 4 Abteilung für Unfallchirurgie, Sportorthopädie und Kindertraumatologie der BG Unfallklinik Murnau, Klinikum Garmisch-Partenkirchen, Murnau am Staffelsee, Deutschland; 5 Berlin, Deutschland; 6 Klinik für Unfallchirurgie und Orthopädie, St. Elisabethen-Klinikum, Ravensburg, Deutschland

**Keywords:** Retrograder ESIN, Konservative Therapie, Korrekturgrenzen, Behandlungsschema, SKT, Retrograde ESIN, Conservative, Correction limits, Treatment regimen, Pediatric traumatology section

## Abstract

**Hintergrund:**

Die proximale Humerusfraktur ist mit 0,45–2 % aller Frakturen eine relativ häufige Verletzung im Kindes- und im Jugendalter [2, 18]. Die Behandlung ist meistens konservativ, aber immer noch Gegenstand der wissenschaftlichen Diskussion [9, 12]. Neben der S1-LL gibt es unterschiedliche Empfehlungen zu Diagnostik und Behandlung dieser Fraktur in der Literatur.

**Methodik:**

Im Rahmen des 10. Wissenschaftstreffens der SKT in der DGU wurden die vorhandenen Empfehlungen und die relevante bzw. aktuelle Literatur kritisch von einem Expertengremium diskutiert und ein Konsens formuliert. In diesen wurde ein Algorithmus zu Diagnostik, Therapie und Behandlung integriert.

**Ergebnisse:**

Die Messung der Achsabweichung und Abkippung ist nicht „interobserver“ und „intraobserver reliable“ [3]. Die Altersgrenze, bis zu der eine vollständige Korrektur möglich ist, wurde auf 10 Jahre festgelegt, da sich ca. um dieses Alter das Korrekturpotenzial ändert. Zur Diagnostik wird die gut zentrierte Röntgenaufnahme in 2 Ebenen (true a.-p.- und Y‑Aufnahme ohne Thoraxanteile) als Standard festgelegt. Im Alter unter 10 Jahren kann jegliche Fehlstellung konservativ mittels Gilchrist-Verband für 2 bis 3 Wochen behandelt werden. Nur in Einzelfällen kann eine Operation z. B. bei starken Schmerzen oder notwendiger rascher Belastbarkeit indiziert sein. Über 10 Jahren sollte ein Ad-latus-Versatz über halbe Schaftbreite nicht toleriert werden. Aufgrund der Varianz der Messergebnisse kann eine Empfehlung zur operativen Versorgung in Abhängigkeit vom Ausmaß der Ad-axim-Dislokation nicht benannt werden. Orientierend gilt: Je größer die Dislokation und je näher das Kind am Fugenschluss ist, desto eher ist die operative Therapie indiziert. Die Entwicklung ist hier einzubeziehen. Den Goldstandard stellt die retrograde, radiale und unilaterale ESIN-Osteosynthese mittels 2 intramedullären Nägeln dar. Die Osteosynthese erfordert keine Ruhigstellung. Ein Verlaufsröntgen ist bei instabilen Frakturen ohne Osteosynthese nach einer Woche, sonst optional zur Dokumentation der Konsolidierung nach 4 (bis 6) Wochen, wenn z. B. die Sportfreigabe erteilt werden soll, sowie vor der Metallentfernung (12 Wochen) vorgesehen.

**Schlussfolgerung:**

Empfehlungen zur Operationsindikation auf der Grundlage des Ausmaßes der Abkippung sind nicht reproduzierbar und erscheinen in Anbetracht der aktuellen Literatur schwierig [3, 9, 12]. Sinnvoller ist ein pragmatisches Vorgehen. Die Prognose der Fraktur erscheint unter Beachtung des erstellten Algorithmus so gut zu sein, dass in den meisten Fällen die Restitutio ad integrum erwartet werden kann.

## Abstract (en)

**Background:**

Proximal humeral fractures are a relatively common injury in childhood and adolescence, accounting for 0.45–2% of all fractures [2, 18]. Treatment is usually conservative but is still the subject of a scientific debate [9, 12]. In addition to the S1-LL, there are different recommendations for the diagnostics and treatment of these fractures in the literature.

**Methodology:**

As part of the 10th scientific meeting of the SKT of the DGU, the existing recommendations and the relevant or current literature were critically discussed by a panel of experts and a consensus was formulated. An algorithm for the diagnostics, therapy and treatment was integrated into this.

**Results:**

The measurement of axial deviation and tilt is not interobserver and intraobserver reliable [3]. The age limit for when complete correction is possible was set at an age of 10 years, as the correction potential changes around this age. For diagnostic purposes, well-centered X‑ray images in 2 planes (true AP and Y‑images without thoracic parts) is defined as the standard. At the age of less than 10 years, any malposition can be treated conservatively with Gilchrist bandaging for 2–3 weeks. Surgery can only be indicated in individual cases, e.g., in the event of severe pain or the need for rapid weight bearing. An *ad latus* displacement of more than half the shaft width should not be tolerated over the age of 10 years. Due to the variance in the measurement results, it is not possible to recommend surgical treatment depending on the extent of the *ad axim* dislocation. As a guideline, the greater the dislocation and the closer the child is to growth joint closure, the more likely surgical treatment is indicated. The development should be taken into account. The gold standard is retrograde, radial and unilateral ESIN osteosynthesis using two intramedullary nails. Osteosynthesis does not require immobilization. A follow-up X‑ray is planned for unstable fractures without osteosynthesis after 1 week, otherwise optional for documentation of consolidation after 4–6 weeks, e.g., if sports clearance is to be granted and before metal removal (12 weeks).

**Conclusion:**

Recommendations for surgical indications based on the extent of tilt are not reproducible and seem difficult in view of the current literature [3, 9, 12]. A pragmatic approach is recommended. The prognosis of the fracture appears to be so good, taking the algorithm into account, that *restitutio ad integrum* can be expected in most cases.

## Einleitung

**Abb. 1:**
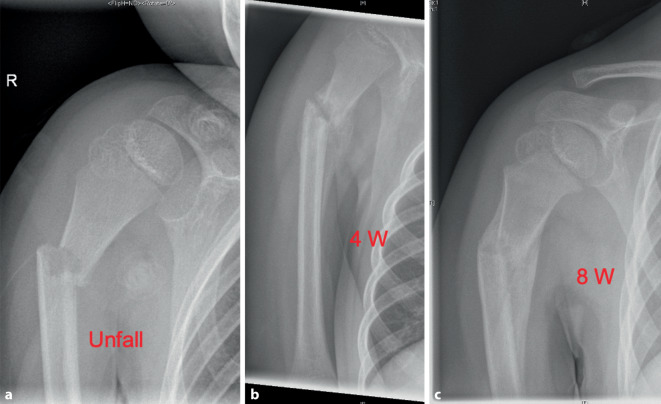
Fünfjähriger Junge nach Sturz beim Fußballspielen. **a** Unfallbild; **b** 4 Wochen nach dem Unfall mit deutlicher Kallusbildung; **c** 8 Wochen nach dem Unfall mit voller Konsolidierung und bereits sichtbarer Korrektur

Die proximale Humerusfraktur (exklusive pathologische Frakturen) ist mit 0,45–2 % aller Frakturen eine relativ häufige Verletzung im Kindes- und im Jugendalter [2, 18]. Der Anteil im Bereich der oberen Extremität ist mit ca. 20 % hoch [16]. Der Altersgipfel liegt bei ca. 10 Jahren [8]. Eine Prävalenz des Geschlechtes ist in der Literatur nicht zu erkennen [2, 8, 12]. Die Frakturen entstehen durch direkte Traumata z. B. im Rahmen von Verkehrsunfällen oder durch indirekte Mechanismen mit Abstützen des ausgestreckten Armes nach hinten bei außenrotierter Schulter (z. B. Stürze von Klettergerüst, Baum oder Pferd) [12]. Im Gegensatz zu den übrigen Frakturen der oberen Extremität gab es in den letzten Jahren trotz des Anstiegs der Risikosportarten keine Zunahme der Verletzung [8]. Begleitverletzungen und Komplikationen, wie z. B. Gefäß‑/Nervenschäden, sind im Vergleich zu anderen Verletzungen, wie z. B. der suprakondylären Humerusfraktur, selten. Die Therapie richtet sich in der gängigen Literatur nach der Dislokation, dem Patientenalter und den Begleitverletzungen [12]. Ziel ist eine funktionelle Restitutio ad integrum unter Toleranz einer vorübergehenden Fehlstellung des proximalen Oberarms. Diese Fraktur hat aufgrund des großen Wachstumspotenzials der proximalen Humerusfuge ein exzellentes Remodeling-Potenzial, insbesondere vor dem 10. Lebensjahr ([19, 23]; Abb. 1).

Die Behandlung sollte meistens konservativ sein, ist aber immer noch Gegenstand der wissenschaftlichen Diskussion [9, 12]. In den letzten Jahren ist trotz zahlreicher Studien, die die große Erfolgsrate der konservativen Therapie zeigten, ein Anstieg der operativen Versorgung zu verzeichnen [8]. Interessant hierbei ist, dass an Krankenhäusern, die sowohl Erwachsene als auch Kinder- und Jugendliche versorgen, die Rate an operativ versorgten Fällen bei 48,2 % lag, während sie an Kinderkrankenhäusern 11,8 % betrug [11, 13]. Akzeptable Toleranzgrenzen unterscheiden sich in der Literatur erheblich. Dislokationen von 20–70° werden hier toleriert [24]. Aufgrund der fehlenden Interobserver- und Intraobserver-Reliabilität der bekannten Messmethoden erscheinen Toleranzgrenzen in Bezug auf die Achsabweichung für die Indikationsstellung jedoch nicht zielführend [3, 9, 12, 26]. Vergleichende oder gar randomisierte Studien liegen nicht vor.

Die konservative Therapie erfolgt, wie im Weiteren beschrieben, mittels Gilchrist-Verband, Tragetuch oder „hanging cast“.

Es sind verschiedene Operationstechniken beschrieben. Mit der retrograden, unilateralen ESIN(Elastisch stabiler intramedullärer Draht)-Osteosynthese von radial hat sich zunehmend eine Operationsmethode mit geringem Risikoprofil und sehr guten Erfolgsergebnissen, bei der keine additive Ruhigstellung notwendig ist, etabliert [29]. Alternativ wird gerade im angloamerikanischen Bereich immer noch die direkte Kirschner-Draht-Osteosynthese beschrieben. Neben der Notwendigkeit einer Ruhigstellung hat sie eine höhere Rate an Komplikationen bzw. sekundären Dislokationen [9].

Eine große Varianz zeigen im Weiteren auch die Nachbehandlung bzw. die Angaben zur Notwendigkeit des Follow-up, inklusive Röntgenverlaufskontrollen [12]. Um sowohl eine pragmatische Indikationsstellung zur konservativen oder zur operativen Therapie als auch eine einheitliche Nachbehandlung zu etablieren, wurden durch den Wissenschaftlichen Arbeitskreis der Sektion Kindertraumatologie (SKT) der Deutschen Gesellschaft für Unfallchirurgie (DGU) im Konsens Behandlungsempfehlungen für die proximale Humerusfraktur im Kindes- und im Jugendalter, die auf der aktuellen Fachliteratur und der Erfahrung der Autoren bzw. der Sektion beruhen, erstellt.

## Zielsetzung

Ziel des Konsensusberichtes ist die Vereinheitlichung von Diagnostik, Therapie und Nachbehandlung der proximalen Humerusfraktur im Kindes- und im Jugendalter. Es sollte ein klarer Algorithmus erstellt werden, der einen für den klinisch Tätigen pragmatischen Ansatz zur Behandlung darstellt und eine Übertherapie vermeidet.

Die proximale Humerusfraktur im Kindes- und im Jugendalter unterscheidet sich zu der von Erwachsenen u. a. durch das Vorhandensein der Wachstumsfuge und das hier besonders große Korrekturpotenzial. Mehrfragmentäre Frakturen stellen eine Rarität dar [15]. Während Fugenverletzungen (Epiphyseolysen der Typen 1 und 2 nach Salter und Harris; AO-PCCF-Typ 11-E/1.1-2.1) gehäuft im Adoleszentenalter auftreten, ist die häufigste Frakturform der kleineren Kinder die subkapitale Humerusfraktur (AO-PCCF-Typ 11-M/2.1-3.1). Diese entsteht aufgrund der metaphysär sehr dünnen Kortikalis, die hier eine Prädilektionsstelle darstellt. Der proximale Humerus besteht aus 2 bis 3 Wachstumskernen, die sukzessive bis zum 5. Lebensjahr auftreten. Sie fusionieren zwischen dem 5. und 7. Jahr. Der Fugenschluss erfolgt ungefähr zwischen dem 14. und dem 17. Lebensjahr (Mädchen) resp. 16. und 18. Lebensjahr (Jungen). Die Behandlungsempfehlung richtet sich somit an alle Kinder und Jugendlichen mit offenen Wachstumsfugen. Pathologische und geburtstraumatische Frakturen sind hierbei auszugrenzen.

## Diagnostik

Bei der klinischen Untersuchung wird der Fokus auf den Erhalt der peripheren Durchblutung, Motorik und Sensibilität gelegt. Gefäß- und Nervenverletzungen sind im Vergleich zu den Erwachsenen eine Rarität. Einzelfallbeschreibungen liegen jedoch vor [12]. In der Inspektion zeigen sich eine Schwellung und ein Hämatom des proximalen Humerus sowie eine meist aufgehobene Beweglichkeit. Spätestens nach Untersuchung der pDMS sollte eine adäquate Analgesie erfolgen.

**Abb. 2:**
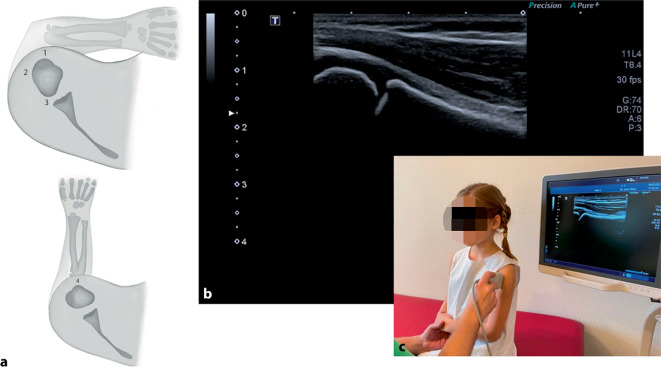
**a** Sonographische Standardschnitte am proximalen Humerus im Piktogramm (*1* Anterior, *2* Lateral, *3* Posterior, *4* Medial). **b** Darstellung des proximalen Humerus (*links* die Epiphyse, *nach rechts auslaufend* der Humerusschaft). **c** Positionierung des Kindes zur schmerzarmen Untersuchung

Besteht der Verdacht auf eine proximale Humerusfraktur, kann als erster Schritt in der Hand des Geübten die Sonographie sinnvoll sein. Nahezu schmerzfrei kann hier der proximale Humerus in 3 bis 4 Ebenen dargestellt und eine Fraktur detektiert werden (Abb. 2). Zumindest zur Vermeidung von Ganzarmaufnahmen aufgrund eingeschränkter Untersuchbarkeit ist die Sonographie zu empfehlen. Die sonographische Detektion einer Fraktur erfordert zumindest ergänzend eine konventionelle radiologische Darstellung in einer Ebene zum Ausschluss einer pathologischen Fraktur [1].

Aus unserer Sicht bleibt die Röntgenaufnahme des proximalen Humerus in 2 Ebenen die Standarddiagnostik. Hierbei ist jedoch dringend darauf zu achten, dass früher übliche transthorakale Aufnahmen aus Gründen der Strahlenhygiene zu vermeiden sind. Die Standardebenen sind hier die true‑a.-p.- und die y‑Aufnahme nach Neer. Sollte nach einer Aufnahme die Therapieentscheidung klar sein, sollte die zweite Aufnahme vermieden werden [14, 27].

Die Schnittbildgebung ist im Bereich der proximalen Humerusfraktur im Kindesalter ohne Stellenwert.

Die Auswertung der Literatur zeigt, dass die häufig angewandte Messung der Abkippung der Fraktur weder Intraobserver- noch Interobserver-Reliabilität bietet und somit für die Indikationsstellung ungeeignet erscheint [6, 7, 21]. Ebenfalls sind die radiologischen Ebenen nicht exakt reproduzierbar, sodass deutliche Messunterschiede der Abkippung entstehen [26].

Eine neuere Methode beschreibt in Anlehnung an die Messung der Epiphyseolysis capitis femoris (ECF) eine gute Interobserver- und Intraobserver-Reliabilität [3]. Hier können laut Angaben der Autoren reproduzierbare Ergebnisse erzielt werden. Empfehlungen in Bezug auf diese Messwinkel und Therapieempfehlungen bestehen jedoch derzeit noch nicht.

## Therapie

**Abb. 3:**
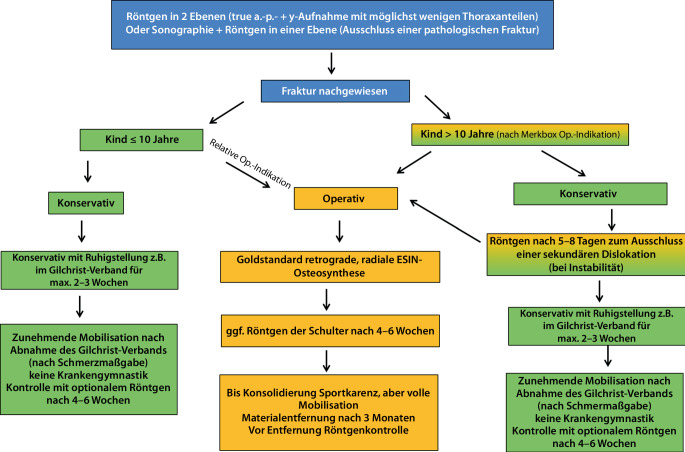
Algorithmus zur Behandlung von proximalen Humerusfrakturen im Kindes- und im Jugendalter

Anhand der mit der Diagnostik gewonnenen Information wird die weitere Therapie geplant. Dabei ist die von uns gewählte Indikationshilfe zur operativen Therapie eher praxisorientiert (Infobox und Abb. 3).

### Infobox Therapieempfehlungen

- Relative Operationsindikationen in allen Altersgruppen: fehlende Akzeptanz der konservativen Maßnahmen, Schmerzen, evtl. frühere Mobilisation, temporär sichtbare kosmetische Beeinträchtigung wird nicht akzeptiert, offene Fraktur, Polytrauma
- 10 bis 14 Jahre: bis > 1/2 Schaftbreite Dislokation, Erwartung der fehlenden Korrektur durch Wachstum, z. B. Tanner-Stadium (Fugenzustand); Stabilität: je mehr Dislokation, umso eher Operation; relative Operationsindikationen (s. oben)

**Abb. 4:**
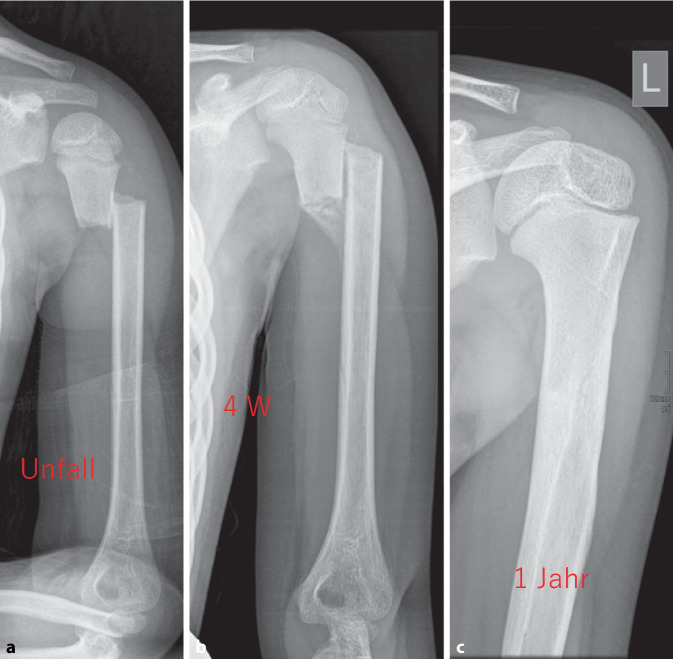
Sechsjähriges Mädchen nach Sturz von der Schaukel; **a** Unfallbild; **b** 4 Wochen nach Unfall; **c** Korrektur ein Jahr nach Trauma

Aufgrund der Veränderung der anatomischen Gegebenheiten und der Variation des Korrekturpotenzials einigten wir uns, die Altersgruppen unter 10 Jahren und über 10 Jahren different zu behandeln. Nach eingehender Recherche der Literatur und einer Meinungsbildung bzw. Fallbegutachtung ist kein Kind unter 10 Jahren beschrieben, welches nach konservativer Therapie keine Restitutio ad integrum im Hinblick auf die Beweglichkeit, Schmerzen oder das optische Erscheinungsbild des Humerus hatte [5, 12, 19, 23]. Die teils nichtkomplette Ausgradung des knöchernen Humerus wird funktionell durch das große Bewegungsspiel des glenohumeralen Gelenks ausgeglichen. So kommen wir zu dem Schluss, dass jedes Kind mit einem knöchernen Alter unter 10 Jahren einer konservativen Therapie zugeführt werden kann (Abb. 4).

Abseits der grundsätzlichen Möglichkeit zur konservativen Therapie bestehen Gegebenheiten, die zu einer fakultativen Operation führen können. Kinder und Jugendliche mit V. a. Gefäß‑/Nervenschaden oder eine Einklemmung der Bizepssehne im Frakturspalt müssen ggf. exploriert werden, und dann empfiehlt es sich auch, selbige zu stabilisieren [20]. Gleiches gilt für polytraumatisierte Patienten und Kinder mit ausgeprägten Schmerzen. Schwache Kriterien für eine Operation sind die ggf. fehlende Akzeptanz der konservativen Maßnahmen, der temporären kosmetischen Abweichung und evtl. der Wunsch nach schneller eintretender Sportfähigkeit bei z. B. Leistungssport.

Da es außer dem Gefäß‑/Nervenschaden und dem Polytraumapatienten keine dringende Operationsindikation gibt, stellt die operative Versorgung der proximalen Humerusfraktur im Kindes- und im Jugendalter nie eine Notfallindikation dar und kann geregelt am Folgetag erfolgen.

### Merke.

Die operative Versorgung der proximalen Humerusfraktur im Kindes- und im Jugendalter stellt bei geschlossenen Weichteilen und intakter pDMS *keine *Notfallindikation dar.

**Abb. 5:**
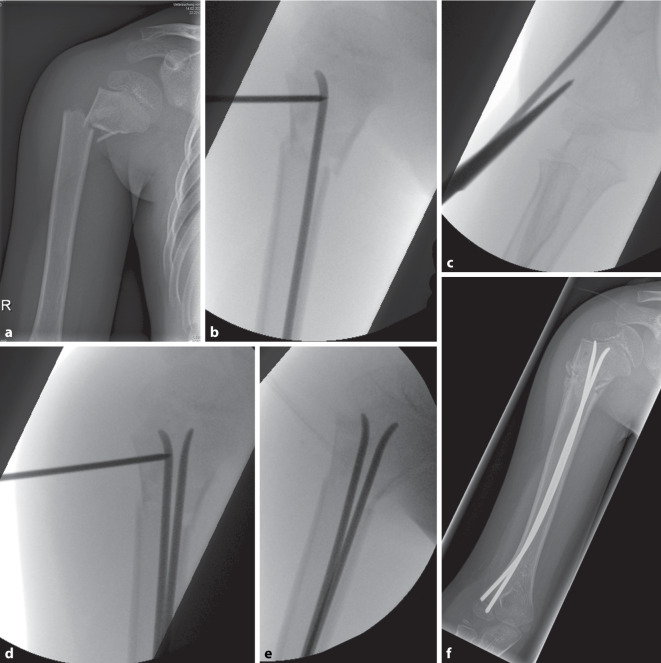
Vierjähriger Junge mit Sturz vom Klettergerüst; Die Eltern waren nicht von einer konservativen Therapie zu überzeugen; Der Junge war sehr schmerzgeplagt; daher ESIN bei lediglich relativer Operationsindikation. **a** Unfallbild; **b** schrittweise Reposition mit Kirschner-Draht zur Fixation des Kopfes; **c** versetzte Eintrittspunkte radial, metaphysär; **d** Positionierung des zweiten Nagels; **e** finale Reposition; **f** postoperatives Röntgen

**Abb. 6:**
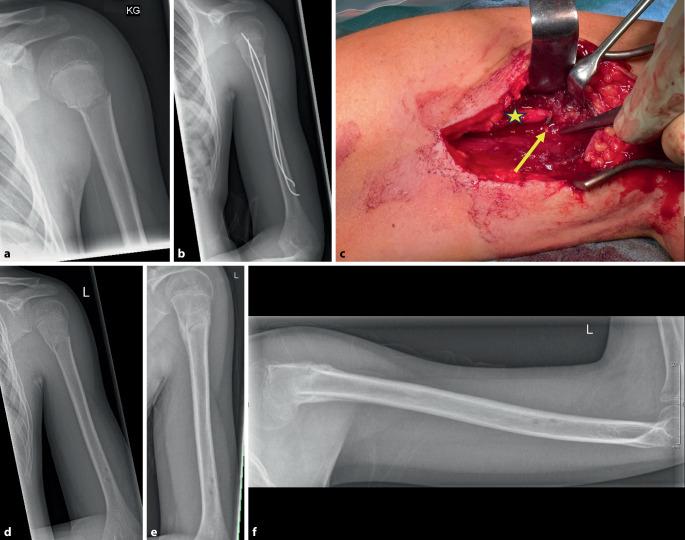
**a** Subkapitale Humerusfraktur eines 13-jährigen Jungen mit geringer Dislokation; **b** extern technisch fehlerhafte retrograde ESIN-Osteosynthese mit sehr proximalem Eintrittspunkt und postoperativer Fallhand sowie sensorischem Ausfall des N. radialis; **c** Revisionsoperation nach 8 Tagen (*gelber Asteriskus*: N. radialis; *gelber Pfeil*: ESIN-Ende, welches den N. radialis einklemmte); **d** postoperative Bilder mit im weiteren konservativer Behandlung; **e** a.-p.-Konsolidierungsbild nach 5 Wochen; **f** Konsolidierungsbild nach 5 Wochen seitlich; die Fallhand war nach 2 Monaten nahezu komplett regredient, die Sensorik komplett wiederhergestellt

Fällt die Indikation zur Operation, ist die Versorgung mit zwei retrograden intramedullären Nägeln von radial inzwischen in Deutschland der Goldstandard (Abb. 5). Diese Operationsmethode minimiert das Risiko eines Schadens des N. axillaris und ist frühfunktionell nachzubehandeln. Wichtig ist, dass das Ziel nicht eine Dreipunktabstützung wie bei der klassischen ESIN-Osteosynthese ist, sondern lediglich eine Fixation des reponierten proximalen Humerus durch die verspreizten Nägel. Die Arbeit von Samara et al. zeigt, dass in Einzelfällen auch ein einzelner Nagel ausreichend seien kann [25]. Dies ist evtl. hilfreich bei Problemen mit der Implantation zweier Nägel bei engem Markraum. Hier empfiehlt sich dann eher, auf einen etwas dickeren Nagel zu wechseln. Ebenfalls muss die regelrechte Implantationsstelle beachtet werden, da bei zu proximalem Einstieg die Gefahr einer Verletzung des N. radialis droht (Abb. 6). Die Implantation sollte nicht über Stichinzisionen erfolgen, sondern nach klarer Darstellung der Humeruskante und einer Kortikalisperforation mit dem Pfriem unter Sicht. Die etwas größere Inzision wird bei der Metallentfernung unumgänglich. Technisch ist zudem darauf hinzuweisen, dass bei sehr instabilen Frakturen der Humeruskopf perkutan mit einem 2 mm Kirschner-Draht angebohrt und gehalten werden kann, um die Reposition und die Nagelplatzierung zu ermöglichen.

Die früher und in einigen Kreisen heute noch propagierte Kirschner-Draht-Osteosynthese hat mehrere Nachteile bei fehlenden Vorteilen im Vergleich mit der ESIN-Osteosynthese. Zwar führt sie nach Studienergebnissen auch zur ausreichenden Ausheilung, jedoch liegt die Komplikationsrate im Vergleich zur intramedullären Nagelung signifikant höher, v. a. hinsichtlich der Infektionsrate, des Repositionsergebnisses und der Verletzung des N. axillaris [10]. Des Weiteren hat sie den großen Nachteil einer notwendigen additiven Ruhigstellung [9]. Aufgrund dieser Argumente, die mit multiplen Studien untermauert sind, wird heutzutage die Kirschner-Draht-Osteosynthese vonseiten der SKT nicht mehr empfohlen werden.

### Merke.

Der Goldstandard zur operativen Versorgung der proximalen Humerusfraktur im Kindes- und im Jugendalter ist die retrograde, unilaterale ESIN-Osteosynthese von radial mit 2 intramedullären Nägeln.

## Nachbehandlung

**Abb. 7:**
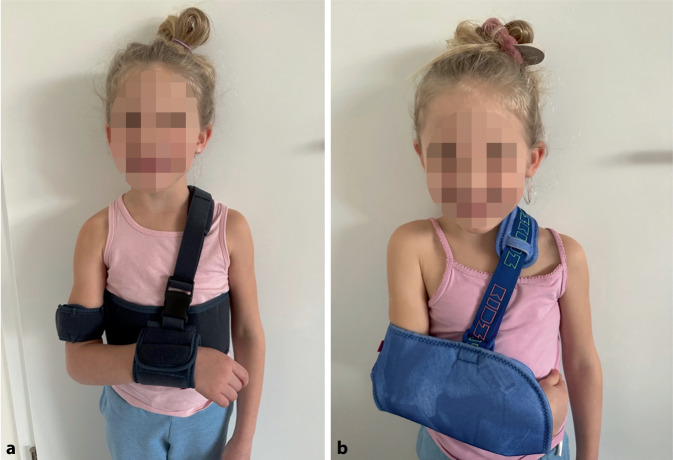
Sechsjährige Zwillingsmädchen mit angelegtem Gilchrist-Verband (**a**) und einfachem Trageverband (**b**)

Fällt die Entscheidung für eine konservative Therapie bei Kindern unter 10 Jahren, so empfehlen wir die Ruhigstellung in Gilchrist-Verband, Tragetuch (Abb. 7) oder äquivalenten Methoden (Desault-Verband, Hanging cast etc.). Der Hanging cast kann bei älteren Kindern mit Grenzindikationen noch eine gewisse Reposition durch den Längszug erreichen [17]. Die Ruhigstellung sollte über 2 bis 3 Wochen angewandt werden. Hiernach kann eine schmerzadaptierte Vollmobilisation erfolgen. Eine Konsolidierung ist mit 4 (bis 6) Wochen zu erwarten. Die Kontrolle kann radiologisch erfolgen, ist aber nicht zwingend erforderlich. In der Praxis hat sich in geübter Hand eine sonographische Kontrolle zu diesem Zeitpunkt als effektiv und mit hoher Akzeptanz gezeigt. Die konservative Therapie der über 10-jährigen Kinder ändert sich lediglich insofern, dass wir bei fraglicher Instabilität eine Stellungskontrolle nach einer Woche und die Konsolidierungskontrolle eher radiologisch empfehlen.

Bei der operativen Behandlung mittels retrogradem ESIN ist keine postoperative Ruhigstellung notwendig. Schmerzadaptiert kann die frühfunktionelle Nachbehandlung erfolgen [29]. Eine Konsolidierungskontrolle kann optional nach 4 (bis 6) Wochen durchgeführt werden. Die Metallentfernung ist nach 12 Wochen möglich; im Vorgang sollte eine radiologische Konsolidierungskontrolle erfolgt sein.

Ob konservativ oder operativ ist eine Sportkarenz bis zur Konsolidierung nach 4 (bis 6) Wochen einzuhalten. Weitere Wachstumskontrollen sind nur bei verbliebenem funktionellem oder ästhetischem Defizit notwendig, dann aber zunächst rein klinisch. Die physiotherapeutische Beübung ist lediglich in Einzelfällen indiziert.

## Prognose

Die Ergebnisse der konservativen Therapie sind sehr gut; Komplikationen sind selten [4, 9, 12, 22, 28]. Aufgrund des hohen Korrekturpotenzials sind relevante bleibende Fehlstellungen kaum zu erwarten oder sie werden durch die 3 Funktionsebenen des Schultergelenks ausgeglichen und sehr gut toleriert. Armlängenunterschiede aufgrund eines vorzeitigen Fugenverschlusses sind die Ausnahme, meist funktionell unbedeutend und fast nie so ausgeprägt, dass ein kosmetisches Problem resultiert [9, 12].

## Fazit für die Praxis

- Die proximale Humerusfraktur hat mit dem distalen Unterarm das größte Korrekturpotenzial im Kindes- und im Jugendalter.
- Bei dieser „gutmütigen“ Fraktur sollte bezüglich der Bildgebung das Prinzip „as low as reasonably achievable“ (ALARA) beachtet werden (Sonographie, Röntgen ohne Thoraxanteile).
- Die Messmethodik ist derzeit nicht zielführend, daher ist ein pragmatischer Therapieansatz notwendig.
- Unter 10 Jahren *kann* jede Fraktur in diesem Bereich konservativ mit sehr gutem funktionellem Ergebnis behandelt werden.
- Über 10 Jahren ist mit steigendem Alter und höherer Dislokation die retrograde, unilaterale, radiale ESIN-Osteosynthese als Goldstandard etabliert.
